# Delayed diagnosis of celiac stenosis causing hepatic transplant ischaemic necrosis: diagnosis by spectral Doppler findings

**DOI:** 10.1259/bjrcr.20150210

**Published:** 2016-10-11

**Authors:** Mark Sun, R Brooke Jeffrey, Michael A DiMaio, Eric W Olcott

**Affiliations:** ^1^Department of Radiology, Stanford University School of Medicine, Stanford, CA, USA; ^2^Department of Pathology, Stanford University School of Medicine, Stanford, CA, USA; ^3^Department of Radiology, VA Palo Alto Health Care System, Palo Alto, CA, USA

## Abstract

Following presentation with abnormal liver function enzymes, confusion and fatigue, a 65-year-old male with alcoholic cirrhosis underwent spectral Doppler sonography that showed tardus parvus-like morphology in the main and left hepatic arteries, although peak systolic velocities and resistive indices remained normal. The patient’s continuing clinical symptoms prompted CT angiography, which demonstrated an unexpected, haemodynamically significant stenosis of the celiac artery. Although the stenosis was successfully stented and the hepatic arterial waveforms normalized, the transplanted liver had already undergone ischaemic necrosis, with resulting failure and the need for retransplantation. Recognition of abnormal waveforms, despite normal peak systolic velocities and resistive indices, with prompt definitive imaging evaluation of the arterial tree beyond just the main hepatic artery, may lead to the diagnosis of unexpected flow-limiting lesions in time to allow revascularization and thus prevent ischaemic transplant failure.

## Clinical presentation and imaging findings

**Figure 1.**
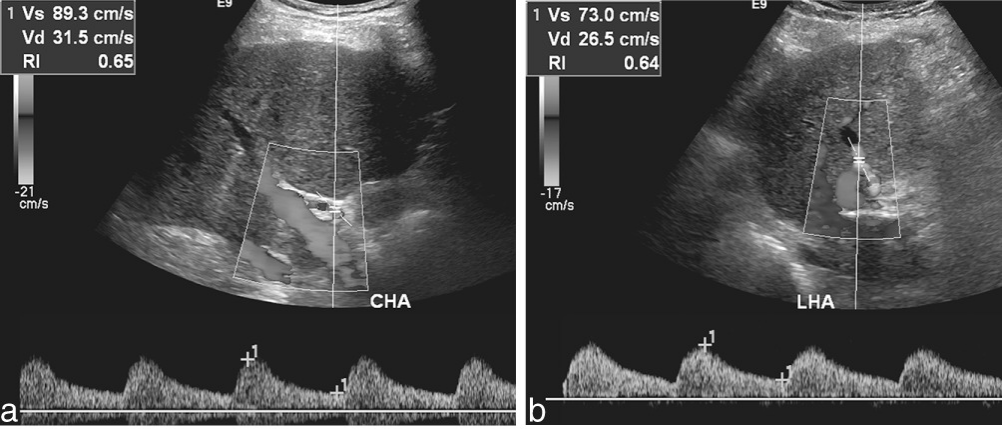
Spectral Doppler tracings at the time of initial presentation show abnormal waveforms with blunting of the systolic upstrokes, although the peak Vs and RI remained normal. (a) Main hepatic artery waveform. (b) LHA waveform. CHA, common hepatic artery; LHA, left hepatic artery; RI, resistive index; Vd, diastolic velocity; Vs, systolic velocity.

**Figure 2.**
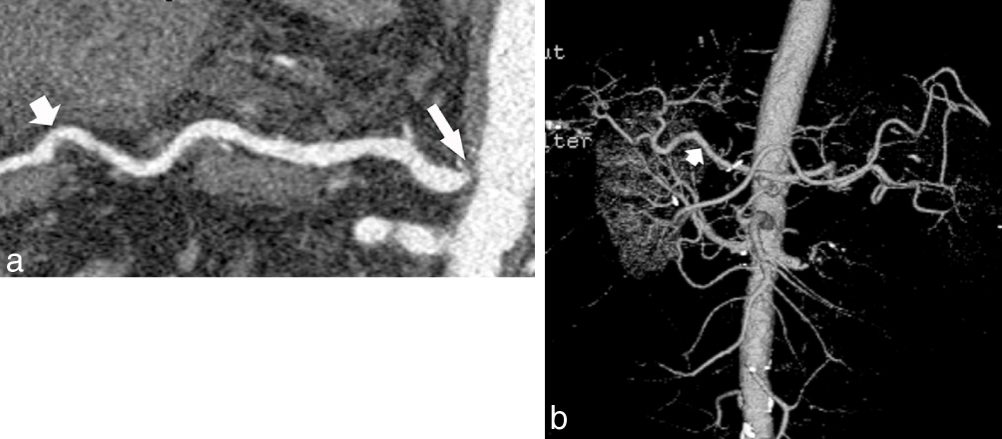
Images from CT angiography. (a) Curved planar reformation, extending from the aorta through the celiac artery and the main hepatic artery, showing a haemodynamically significant celiac artery stenosis (long arrow) and no stenosis in the region of the surgical anastomosis in the main hepatic artery (short arrow). No arterial calcification is evident. (b) Volume rendered image demonstrates the transplant hepatic artery (arrow) and the visceral vasculature. No significant collateral vessels are present. The celiac artery stenosis is not well visualized in this frontal projection.

A 65-year-old male with a history of alcoholic cirrhosis and orthotopic liver transplantation presented with elevated liver function enzymes, confusion and fatigue. The transplantation procedure, performed 11 years ago, had been conducted in the standard fashion, with venovenous bypass, ligation of the patient’s gastroduodenal artery and end-to-end anastomosis between the donor’s celiac artery and the patient’s common hepatic artery. The patient had been taking medication for hypertension and had a 10year history of cigarette use. Spectral Doppler sonography demonstrated abnormal waveforms with tardus parvus-like morphology within the main and left hepatic arteries, both of which demonstrated blunting of the systolic upstroke (Figure 1a). However, unlike the classic tardus parvus pattern, the peak systolic velocities remained within normal limits (measuring approximately 70–90 cm s^–1^) and the resistive indices (RIs) also remained within normal limits, not falling below 0.5 (measuring 0.65 and 0.64 in the main and left hepatic arteries, respectively).^1^ Despite these normal values, the blunted systolic upstroke and the patient’s unremitting clinical presentation prompted concern for a flow-limiting arterial lesion causing graft ischaemia. CT angiography 2 days thereafter revealed not only a normal main hepatic artery and patent surgical arterial anastomosis, but also an unexpected, haemodynamically significant stenosis (exceeding 50% diameter^2^) at the origin of the celiac artery (Figure 2).

## Treatment and outcome

The patient underwent transarterial celiac angiography 2 days after the CT angiogram and 4 days following his initial clinical presentation and sonogram. A balloon-expandable stent was successfully placed across the celiac lesion, with resolution of the stenosis and improved, robust flow throughout the hepatic arterial tree.

**Figure 3.**
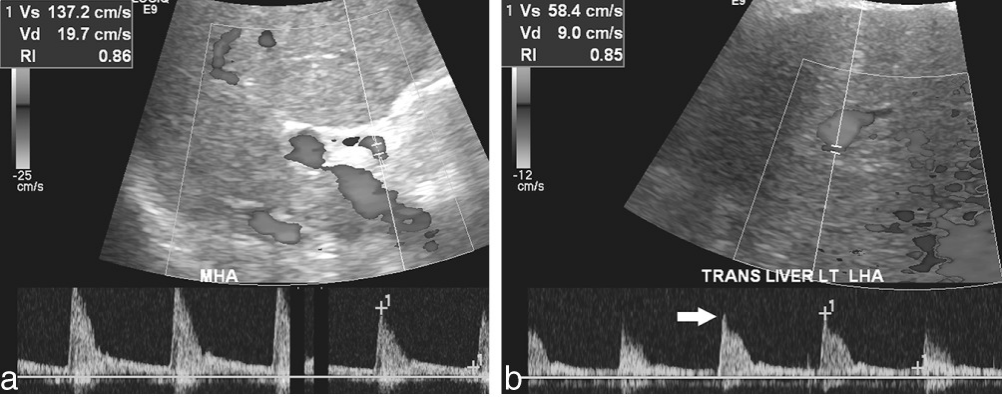
Spectral Doppler tracings after celiac artery stenting demonstrate improved waveforms but persistently elevated RIs. (a) The MHA and (b) the LHA waveforms now show normal, brisk systolic upstrokes, with restitution of the early systolic peak in the latter (arrow). Additionally, the peak Vs is subnormal in the LHA. LHA, left hepatic artery; MHA, main hepatic artery; RI, resistive index; Vd, diastolic velocity; Vs, systolic velocity.

Follow-up hepatic artery Doppler sonography 2 days later demonstrated normalization of the hepatic arterial waveform morphology in the main and left hepatic arteries, with restitution of brisk upstrokes in both and restitution of the early systolic peak in the latter (Figure 3). However, the RIs did not diminish as would be expected, remaining elevated at approximately 0.86, and the peak systolic velocity in the left hepatic artery was subnormal at 58 cm s^–1^. These findings prompted transjugular liver biopsy, which demonstrated extensive ischaemic necrosis within the transplanted liver. Ultimately, owing to the unabated and severe hepatic dysfunction caused by the ischaemia, the transplant was declared irretrievable and the patient was scheduled to undergo retransplantation.

Despite all available efforts, the patient could not be contacted and thus informed consent could not be obtained; however, no identifying personal information is presented herein.

## Discussion

Hepatic arterial complications are a major source of morbidity and mortality following liver transplantation and can occur with grafts from non-living as well as living donors.^1–4^ These complications usually occur at the site of donor/recipient anastomosis and can include thrombosis, stenosis, dissection, arteriovenous fistulas and pseudoaneurysm formation. Spectral Doppler sonography is the mainstay imaging modality for monitoring post-operative hepatic blood flow and, accordingly, the early detection of arterial complications. The classic tardus parvus waveform, for example, with diminished systolic upstroke slope and RI < 0.5, has been shown to be both sensitive and specific for upstream arterial lesions within the hepatic artery.^1–4^ Correct diagnosis of flow-limiting lesions is crucial to permit prompt revascularization, when necessary, to prevent ischaemic necrosis and loss of grafted livers.^1,5,6^ In the acute setting, diminished arterial inflow may lead to graft infarction and insufficiency, and in the subacute or chronic setting, it may result in biliary necrosis. Retransplantation is often the only effective course of therapy for a graft that has become extensively infarcted, and non-invasive monitoring of hepatic arterial blood flow with spectral Doppler sonography is critically important for the early detection of hepatic arterial insufficiency.^1,5^

Less frequently, but no less importantly, diminished hepatic arterial inflow may relate to lesions in the celiac artery rather than the anastomosis within the hepatic artery. Additionally, diminished venous outflow may impede hepatic arterial inflow. Park et al^6^ have noted that the tardus parvus waveform can be seen in patients with celiac artery stenosis, post-biopsy arterioportal fistulas and flow-limiting lesions of the portal or hepatic veins. We present a case of a hepatic transplant compromised by an unexpected celiac artery stenosis, a lesion that was not diagnosed promptly enough to prevent ischaemic necrosis and loss of the graft.

This case illustrates several essential points relevant to the sonographic evaluation of a transplanted liver. The first is the importance of recognizing that an abnormal hepatic arterial waveform may indicate significant inflow restriction even when other values, such as the peak systolic velocity and the RI, remain within normal limits, which they did in this patient. Not every patient with significant flow-limiting lesions demonstrates classic tardus parvus findings.^5^ Indeed, the classic finding of RI <  0.5 was not observed in this case, in spite of the significant celiac artery stenosis. We speculate that this unusual occurrence reflects increased resistance within the transplant arterial bed as the graft was undergoing ischaemic necrosis, supporting the elevated RI values.

Second, timely imaging evaluation is essential in the setting of a possibly ischaemic transplant. 4 full days passed between the patient’s presentation with tardus parvus-like hepatic arterial waveforms and the stenting procedure that finally restored robust hepatic arterial flow. We speculate that had more rapid intervention been performed, the extent of ischaemia may have been limited and the graft failure averted. With this in mind, definitive imaging (CT, MR or transarterial angiography) should be pursued promptly when an abnormal hepatic arterial waveform is recognized in the setting of graft dysfunction.

It is essential to consider feeding vessels in addition to the main hepatic artery itself, such as the celiac artery, when searching for flow-limiting lesions. In the present case, the haemodynamically significant celiac stenosis undoubtedly developed after the patient’s original, successful transplant surgery. Because no calcifications were demonstrated at the site of the celiac stenosis by CT angiography, the differential diagnosis would include non-calcified atherosclerotic plaque as well as the median arcuate ligament syndrome.^7–9^ Some authors have noted that celiac artery compression by the arcuate ligament occurs in as many as 10% of patients undergoing liver transplantation.^7^ It is rarely symptomatic, owing to efficient arterial collateralization from the superior mesenteric and the gastroduodenal arteries; however, in patients without sufficient collateralization, symptomatic hepatic arterial insufficiency and/or thrombosis may result. Fortunately, the stenting procedure for our patient restored flow such that, for either possible cause, the patient’s retransplantation can proceed with adequate arterial inflow.

In summary, abnormal hepatic arterial waveforms following hepatic transplantation, even when other values such as RIs and peak systolic velocities are not abnormal, should elicit a prompt search for flow-limiting lesions using definitive imaging modalities such as CT angiography, with careful evaluation for lesions not only at the arterial anastomosis but also at other sites within the arterial tree supplying the transplanted liver.

## Learning points

1. Abnormal hepatic arterial waveforms in hepatic transplant patients, despite normal peak systolic values and normal RIs, should elicit a search for flow-limiting arterial lesions.
2. Definitive imaging, such as CT angiography, should be pursued promptly in the search for flow-limiting arterial lesions.
3. Severe complications following liver transplantation may result from unexpected flow-limiting arterial lesions at sites other than the typical location at the donor/recipient anastomosis.

## Consent

Our institutional review board approved this study and waived informed consent in view of its retrospective nature. All procedures were conducted in compliance with the Health Insurance Portability and Accountability Act.

## References

[1] Dani G, Sun MR, Bennett AE. Imaging of liver transplant and its complications. Semin Ultrasound CT MR 2013; 34: 365–77. 23895908 10.1053/j.sult.2013.04.002

[2] Dodd GD, Memel DS, Zajko AB, Baron RL, Santaguida LA. Hepatic artery stenosis and thrombosis in transplant recipients: Doppler diagnosis with resistive index and systolic acceleration time. Radiology 1994; 192: 657–61. 8058930 10.1148/radiology.192.3.8058930

[3] Nghiem HV, Tran K, Winter TC, Schmiedl UP, Althaus SJ, Patel NH, et al Imaging of complications in liver transplantation. Radiographics 1996; 16: 825–40. 8835974 10.1148/radiographics.16.4.8835974

[4] Vit A, De Candia A, Como G, Del Frate C, Marzio A, Bazzocchi M. Doppler evaluation of arterial complications of adult orthotopic liver transplantation. J Clin Ultrasound 2003; 31: 339–45. 12923877 10.1002/jcu.10190

[5] Choi EK, Lu DS, Park SH, Hong JC, Raman SS, Ragavendra N Doppler US for suspicion of hepatic arterial ischemia in orthotopically transplanted livers: role of central versus intrahepatic waveform analysis. Radiology 2013; 267: 276–84. 23297323 10.1148/radiol.12120557

[6] Park YS, Kim KW, Lee SJ, Lee J, Jung DH, Song GW, et al Hepatic arterial stenosis assessed with doppler US after liver transplantation: frequent false-positive diagnoses with tardus parvus waveform and value of adding optimal peak systolic velocity cutoff. Radiology 2011; 260: 884–91. 21734158 10.1148/radiol.11102257

[7] Jiang ZJ, Liang TB, Feng XN, Wang WL, Shen Y, Zhang M, et al Arcuate ligament syndrome inducing hepatic artery thrombosis after liver transplantation. Hepatobiliary Pancreat Dis Int 2008; 7: 433–6. 18693183

[8] Jurim O, Shaked A, Kiai K, Millis JM, Colquhoun SD, Busuttil RW. Celiac compression syndrome and liver transplantation. Ann Surg 1993; 218: 10–12. 8328823 10.1097/00000658-199307000-00003 PMC1242894

[9] Vilatobá M, Zamora-Valdés D, Guerrero-Hernández M, Romero-Talamás H, Leal-Villalpando RP, Mercado MA Arcuate ligament compression as a cause of early-onset thrombosis of the hepatic artery after liver transplantation. Ann Hepatol 2011; 10: 88–92. 21301017

